# A “Smart” Force-Limiting Instrument for Microsurgery: Laboratory and In Vivo Validation

**DOI:** 10.1371/journal.pone.0162232

**Published:** 2016-09-13

**Authors:** Hani J. Marcus, Christopher J. Payne, Ahilan Kailaya-Vasa, Sara Griffiths, James Clark, Guang-Zhong Yang, Ara Darzi, Dipankar Nandi

**Affiliations:** 1 The Hamlyn Centre, Institute of Global Health Innovation, Imperial College London, London, United Kingdom; 2 Department of Neurosurgery, Imperial College Healthcare NHS Trust, London, United Kingdom; 3 Department of Neurosurgery, Barking, Havering and Redbridge University Hospitals NHS Trust, Essex, United Kingdom; Universita degli Studi di Napoli Federico II, ITALY

## Abstract

Residents are required to learn a multitude of skills during their microsurgical training. One such skill is the judicious application of force when handling delicate tissue. An instrument has been developed that indicates to the surgeon when a force threshold has been exceeded by providing vibrotactile feedback. The objective of this study was to validate the use of this “smart” force-limiting instrument for microsurgery. A laboratory and an *in vivo* experiment were performed to evaluate the force-limiting instrument. In the laboratory experiment, twelve novice surgeons were randomly allocated to use either the force-limiting instrument or a standard instrument. Surgeons were then asked to perform microsurgical dissection in a model. In the *in vivo* experiment, an intermediate surgeon performed microsurgical dissection in a stepwise fashion, alternating every 30 seconds between use of the force-limiting instrument and a standard instrument. The primary outcomes were the forces exerted and the OSATS scores. In the laboratory experiment, the maximal forces exerted by novices using the force-limiting instrument were significantly less than using a standard instrument, and were comparable to intermediate and expert surgeons (0.637N versus 4.576N; p = 0.007). In the *in vivo* experiment, the maximal forces exerted with the force-limiting instrument were also significantly less than with a standard instrument (0.441N versus 0.742N; p <0.001). Notably, use of the force-limiting instrument did not significantly impede the surgical workflow as measured by the OSATS score (p >0.1). In conclusion, the development and use of this force-limiting instrument in a clinical setting may improve patient safety.

## Introduction

Mastering the art of microsurgery is a long and challenging endeavor. Surgeons typically gain operative expertise through almost a decade of training during residency and fellowship before refining their technique in independent practice. Recent working hour limits imposed on residents have reduced their caseload, with implications on resident education and patient safety[1]. Improvements in training quality, including the use of surgical simulation, may somewhat mitigate the effects of resident duty hour restrictions[2, 3]. In addition to advances in resident education, the development of new surgical devices may play a role in reducing the learning curve, particularly for technically demanding procedures.

A key skill that residents must learn during their training is the judicious handling of delicate tissue. Sufficient force must be applied to successfully carry out microsurgical maneuvers and economize movements, but undue force can result in injury. Recent studies have quantified forces exerted in microsurgery, demonstrating a magnitude that is typically less than 1N[4–6]. Novices exert greater force than experts that leads to an increased risk of causing iatrogenic injury[7].

The advent of robot-assisted surgery has allowed the continuous measurement of instrument forces during a procedure. This allows for the possibility of force limits to be set so as to improve safety. However, most surgical robots used today remain large, complex, and expensive. A different approach in harnessing advanced technology is the use of small, simple, and inexpensive, “smart” microsurgical instruments[8].

A hand-held microsurgical instrument has been developed that indicates to the surgeon when a force threshold has been exceeded by providing vibrotactile feedback[9]. The initial study validated the use of this instrument in a low-fidelity laboratory and an *ex vivo* cadaver experiment[9]. The aim of this study was to extend this work and assess the use of this instrument in a high-fidelity laboratory and an *in vivo* porcine experiment.

## Methods

### Device

**Fig 1:**
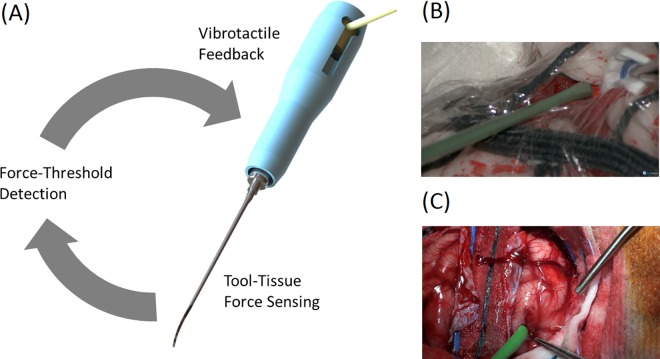
(A) The force-limiting instrument concept. (B) Instrument in use during the laboratory experiment demonstrating the middle cerebral artery aneurysm. (C) Instrument use during the *in vivo* experiment.

A detailed description of the development and design of the force-limiting instrument has previously been reported[9]. We present a new implementation of the device that incorporates 3 axes of force sensing. In brief, the instrument is a modification of a blunt surgical dissector (Yasargil FD304R, B. Braun, Melsungen, Germany). It has been engineered to incorporate a force sensor (Nano17, ATI Industrial Automation, North Carolina, USA) capable of measuring loads applied to its tip, and an on-board actuator to generate subtle vibrations when a force threshold has been breached (Fig 1). The force limit was set to 0.3N, as previously studies have suggested that forces beyond this threshold may risk iatrogenic injury[5].

A toggle was able switch between use of the modified dissector as a force-limiting instrument (forces recorded, and feedback provided when threshold breached), or a standard instrument (forces recorded only).

### Laboratory experiment

#### Participants and study settings

Twelve novices (<10 microsurgical procedures), and six intermediates or experts (10–50 and >50 microsurgical procedures respectively), were recruited from a university hospital. It was decided *a priori* that intermediate and experts surgeons would be combined for subsequent analysis. Informed written consent was obtained from all surgeons.

All surgeons underwent a training session to familiarize themselves with the instruments and the tasks.

#### Trial design

A preclinical randomized study design was adopted, comparing the force-limiting instrument against standard instruments.

A preclinical brain model of the trans-Sylvian approach (Kezlex, Ono & Co., Tokyo, Japan) was utilized to simulate arachnoid dissection (Fig 1). The model was prepared using existing models of the skull, brain, and cerebral vasculature. Acrylic was used to paint small arteries onto the cerebral surface, and to represent a right middle cerebral artery (M1/M2) aneurysm. Synthetic and cotton fibers were use to fashion the Sylvian vein. Polyvinylidene chloride film was used to represent the arachnoid membrane, and wet water-insoluble tissue paper for the arachnoid trabeculae. Previous studies have demonstrated this model has face and content validity[10].

An operating microscope (Leica, Solms, Germany) was used for visualization, and the video feed recorded. A standard set of microsurgical instruments including forceps, scissors, and dissectors, were used in the surgeon’s dominant hand. The modified dissector was used in the surgeon’s non-dominant hand to provide dynamic retraction.

To compare force-limiting against standard instruments, twelve novice surgeons were randomly allocated to use the modified dissector either as a force-limiting instrument or a standard instrument. Surgeons were then asked to perform arachnoid dissection to expose the aneurysm. The task was considered complete once surgeons identified the middle cerebral artery aneurysm. To provide construct validity for the preclinical brain model, six intermediate or expert surgeons also performed the task using the modified dissector as a standard instrument.

#### Outcomes

The primary outcomes were the forces exerted and the modified Objective Structured Assessments of Technical Skill (OSATS) scores. The forces exerted included the median force, the maximum force, and the time spent above the force limit of 0.3N. The OSATS scoring criteria include economy of movement (time and motion), confidence of movement (instrument handling), respect for tissue, and precision of operative technique (flow of operation).

Two data analysts independently reviewed operative videos using the above criteria. Whereas surgeons were aware of whether they were using a force-limiting or standard instrument, the data analysts were blinded to their allocation.

#### Statistical analysis

The sample size was calculated on the basis of a pilot study using the above methodology. It was estimated that to detect a fall in the maximal force exerted by novices using a standard instrument from 5±2.5N to 1N, with a two-sided 5% significance level and a power of 80%, a sample size of at least 6 surgeons was necessary in each group.

Data was analyzed with SPSS v 22.0 (IBM, Illinois, USA). The median and interquartile ranges were calculated for all outcome measures. To assess the construct validity of the preclinical brain model, nonparametric tests were performed comparing novices versus intermediates and experts using the modified dissector as a standard instrument. To assess the force-limiting instrument, nonparametric tests (Mann-Whitney U Test) were then performed comparing novices using the modified dissector as a standard versus force-limiting instrument. A value of p < 0.05 was considered statistically significant.

### In vivo experiment

The study protocol was approved by the home office under the Home Office Animals for Scientific Procedure Act 1986 (No. 80/2297).

#### Participants and study settings

An intermediate surgeon was recruited from a university hospital. Informed written consent was obtained.

#### Trial design

A preclinical alternating study design was adopted, comparing the force-limiting instrument against standard instruments.

An adult white Landrace female pig (70kg) was anaesthetized with intramuscular ketamine and xylazine. After endotracheal intubation, anesthesia was maintained with oxygen, nitrous oxide, and isoflurane, and at the conclusion of the procedure the animal was terminated with sodium pentobarbitone.

The pig was positioned prone with their head extended and secured in a clamp. A custom image guidance platform was used to plan the operative approach. A bicoronal incision was made, and a high-speed drill (B. Braun, Melsungen, Germany) used to fashion a bifrontal craniotomy. A durotomy was performed and dural flaps pedicled over the sinus.

An operating microscope (Zeiss, Oberkochen, Germany) was used for visualization, and the video feed recorded. A standard set of microsurgical instruments were used in the surgeon’s dominant hand, and the modified dissector in the non-dominant hand. Microsurgical dissection proceeded in a stepwise fashion, alternating every 30 seconds between use of the dissector as a standard and force-limiting instrument. A total of 12 minutes of dissection was performed, comprising 6 minutes as a standard instrument and 6 minutes as a force-limiting instrument.

#### Outcomes

The primary outcomes were the forces exerted and the modified OSATS scores, as in the above laboratory experiment.

#### Statistical analysis

The median and interquartile ranges were calculated for all outcome measures of each 30 second period. To assess the force-limiting instrument, nonparametric tests (Mann-Whitney U Test) were performed comparing use of the modified dissector as a standard versus force-limiting instrument. A value of p < .05 was considered statistically significant.

## Results

### Laboratory experiment

The median age of participants was 22 years (range 22 to 37 years), the male:female ratio was 2:1, and the right:left handedness ratio was 5:1. The detailed demographics of the participants are summarized in Table 1. All participants that were enrolled completed the study, and no losses occurred after randomization.

**Table 1:** Participant demographics.

	Age	Sex	Handedness
	Median (range), years	Male:Female	Right:Left
Novice, n = 12	22 (22–23)	6:6	12:0
Intermediate, n = 5	32 (31–36)	5:0	3:2
Expert, n = 1	37	1:0	0:1
Overall, n = 18	22 (22–37)	12:6	15:3

The median and interquartile ranges of performance are summarized in Table 2. The OSATS scores by intermediates and experts using the standard instrument were significantly greater than novices using the standard instrument (26.5 versus 10.8; p = .004). Notably, the maximal forces exerted by intermediates and experts were significantly less than by novices (0.793N versus 4.576N; p = .025) providing construct validity for this metric.

**Table 2:** Laboratory performance of: novice surgeons versus intermediate and expert surgeons using the standard instrument; and novice surgeons using the standard versus force-limiting instrument.

	Median Force	Maximum Force	Time >0.3N	OSATS
	Median (interquartile range), N	Median (interquartile range), N	Median (interquartile range), s	Median (interquartile range)
Novice (standard instrument), n = 6	0.236 (0.143–0.913)	4.576 (2.100–6.869)	96.9 (49.2–280.4)	10.8 (10.0–12.0)
Intermediate and expert (standard instrument), n = 6	0.157 (0.116–0.303)	0.793 (0.511–1.704)	38.5 (1.05–85.3)	26.5 (26.0–27.0)
	p = .47	p = .025*	p = .11	p = .004*
Novice (force-limiting instrument), n = 6	0.121 (0.104–0.143)	0.637 (0.531–0.739)	8.8 (5.8–12.5)	11.8 (11.0–13.0)
	p = .066	p = .007*	p = .004*	p = .30

* p < 0.05

**Fig 2:**
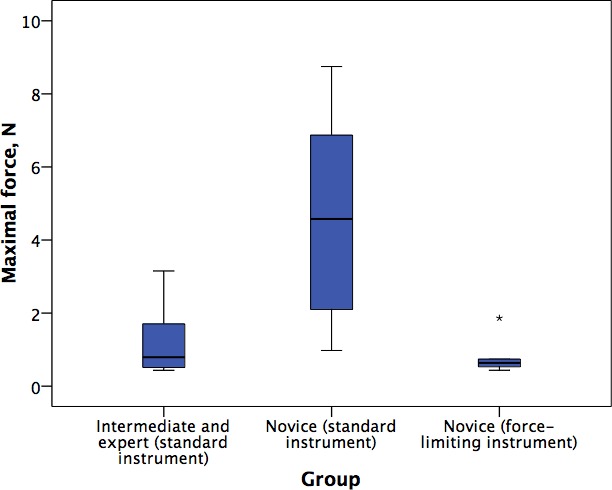
Maximum forces exerted by: intermediates and experts using the standard instrument; novices using the standard instrument; and novices using the force-limiting instrument. Points represent outliers (star greater than 3 times the interquartile range).

The maximal forces exerted by novices using the force-limiting instrument were significantly less than using the standard instrument (0.637N versus 4.576N; p = .007). Indeed, the maximal forces exerted by novices using the force-limiting instrument were comparable to intermediates and experts using the standard instrument (Fig 2). The OSATS scores of novices using the force-limiting instrument were not significantly different to those using the standard instrument (11.8 versus 10.8; p = .30).

### In vivo experiment

**Fig 3:**
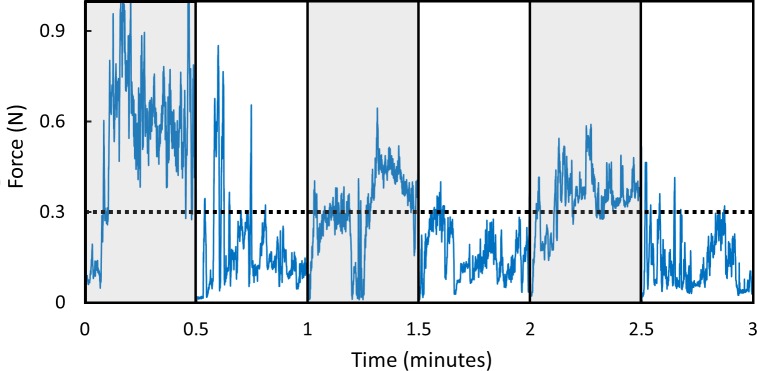
Force over time for the initial six 30 second periods of use during the *in vivo* study. Use of the standard instrument is denoted by a grey background and use of the force-limiting instrument with a white background.

A 32 year-old right-handed male surgical trainee completed the study. The force over time for the initial six 30 second periods are illustrated in Fig 3. The median and interquartile ranges of performance are summarized in Table 3, and were comparable to the performance of intermediate and expert surgeons in the aforementioned laboratory experiment. The maximal forces exerted with the force-limiting instrument were significantly less than with the standard instrument (0.441N versus 0.742N; p < .001). The OSATS scores when using the force-limiting instrument were not significantly different to those using the standard instrument (p = .746).

**Table 3:** *In vivo* performance of an intermediate surgeon using the standard versus force-limiting instrument.

	Median Force	Maximum Force	Time >0.3N	OSATS
	Median (interquartile range), N	Median (interquartile range), N	Median (interquartile range), s	Median (interquartile range)
Single intermediate subject (standard instrument), n = 12	0.319 (0.221–0.403)	0.742 (0.618–0.816)	16.6 (10.9–21.8)	24 (24.0–25.0)
Single intermediate subject (force-limiting instrument), n = 12	0.148 (0.132–0.161)	0.441 (0.394–0.488)	1.2 (0.9–1.6)	24 (24.0–25.0)
	p = .004*	p < .001*	p = .002*	p = .75

* p < 0.05

## Discussion

### Principal findings

Smart instruments represent a novel approach to enhancing a surgeon’s abilities. In this study it has been demonstrated that the use of a force-limiting instrument can effectively limit the maximal forces exerted by novice surgeons, in a validated high-fidelity laboratory model. Moreover, it has been shown that these findings are generalizable to intermediate surgeons, and to an *in vivo* setting. Notably, use of the force-limiting instrument did not significantly impede the surgical workflow as measured by the OSATS score; indeed, there was a trend towards improved performance with the force-limiting instrument.

### Comparison with other studies

Several mechatronic devices that provide force feedback during microsurgery have previously been described. Master-slave platforms, such as those by Sutherland *et al* and Salcudean *et al*, allow for force feedback and can therefore reduce the forces exerted by surgeons[11, 12]. Cooperative platforms, such as the Steady Hand robot, are arranged such that the surgeon and robot share control of instruments, and can also provide force scaling[13]. Hand-held platforms that are unattached to grounded robotic linkages represent small, simple, and inexpensive, alternatives to these master-slave and cooperative platforms. Although hand-held platforms incorporating force sensing have been reported in the biomedical engineering literature[14–16], to the best of our knowledge the experiments presented here are the first attempt at objective evaluation of these devices using validated preclinical models.

### Limitations

The modified dissector used in this study represents a single prototype device. Further development might allow for a range of force-limiting instruments. In bimanual tasks the use of such force-limiting instruments with vibrotactile feedback would confer a considerable advantage over e.g. auditory feedback, allowing the surgeon to intuitively and unambiguously identify which instrument has breached the force threshold.

Intermediate and expert surgeons were combined for analysis in the laboratory experiment due to lack of experts familiar with these uncommon and difficult approaches, and because validation of the model was not the primary aim of the study. Nonetheless, the finding that the maximal force exerted by intermediates and experts was significantly less than by novices, supports use of this outcome measure.

A single intermediate surgeon was recruited for the *in vivo* experiment due to the high cost and logistical difficulties of arranging such a study. In any case, the forces exerted by the intermediate surgeon *in vivo* were comparable to those exerted by intermediate and expert surgeons in the laboratory experiment, and use of the force-limiting instrument effectively reduced the maximal forces.

### Conclusions

In conclusion, the laboratory and an *in vivo* experiment above constitute strong preclinical evidence for the use of the force-limiting instrument to reduce the forces exerted by trainee surgeons. The development and use of this force-limiting instrument in a clinical setting may improve patient safety.

## Supporting Information

S1 Fig Force over time during the entire *in vivo* study. Use of the standard instrument is denoted by a grey background and use of the force-limiting instrument with a white background.
(TIFF)
