# Supplementary figures and images for: A “Smart” Force-Limiting Instrument for Microsurgery: Laboratory and In Vivo Validation
Source: PLoS One. 2016 Sep 13;11(9):e0162232. doi: 10.1371/journal.pone.0162232 (PMC5021258; doi:10.1371/journal.pone.0162232)

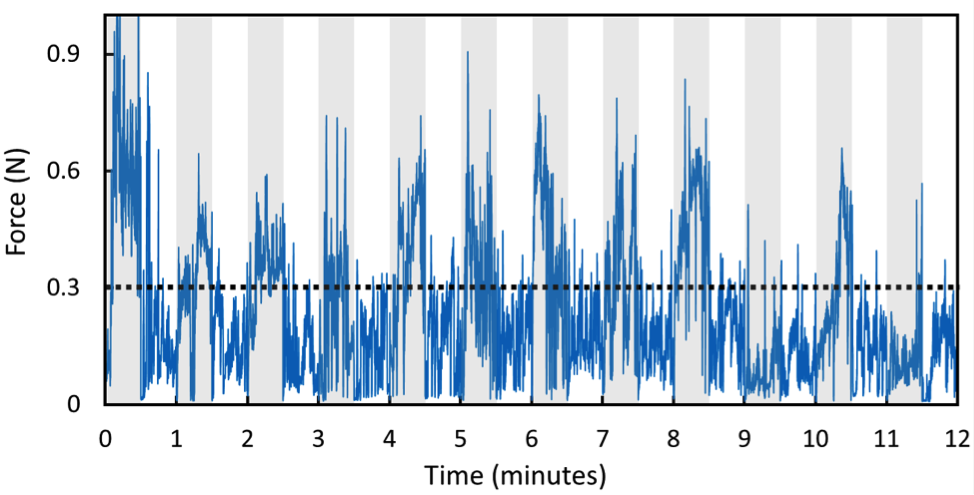

Supplement: S1 Fig — Use of the standard instrument is denoted by a grey background and use of the force-limiting instrument with a white background. (TIFF) [file pone.0162232.s001.tiff]
